# Numerical Model Validation of the Blood Flow through a Microchannel Hyperbolic Contraction

**DOI:** 10.3390/mi14101886

**Published:** 2023-09-30

**Authors:** Filipe Barbosa, Jorge Dueñas-Pamplona, Cristiano S. Abreu, Mónica S. N. Oliveira, Rui A. Lima

**Affiliations:** 1 Mechanical Engineering and Resource Sustainability Center (METRICS), University of Minho, 4800-058 Guimarães, Portugal; a88077@alunos.uminho.pt; 2 Departamento de Ingeniería Energética, Universidad Politécnica de Madrid, 28040 Madrid, Spain; jorge.duenas.pamplona@upm.es; 3 Center for MicroElectromechanical Systems (CMEMS-UMinho), University of Minho, 4800-058 Guimarães, Portugal; csa@isep.ipp.pt; 4 LABBELS—Associate Laboratory, 4710-057 Braga, Portugal; 5 Physics Department, Porto Superior Engineering Institute, ISEP, 4200-072 Porto, Portugal; 6 James Weir Fluids Laboratory, Department of Mechanical and Aerospace Engineering, University of Strathclyde, Glasgow G1 1XJ, UK; monica.oliveira@strath.ac.uk; 7 CEFT—Transport Phenomena Research Center, Faculty of Engineering, University of Porto, 4200-465 Porto, Portugal; 8 ALiCE—Associate Laboratory in Chemical Engineering, Faculty of Engineering, University of Porto, 4200-465 Porto, Portugal

**Keywords:** blood flow, hyperbolic contraction, experimental validation, numerical modeling, microfluidics

## Abstract

A computational fluid dynamics (CFD) model of blood flow through hyperbolic contraction with a discrete phase model (DPM) was experimentally validated. For this purpose, the positions and velocities of red blood cells (RBCs) flowing in a microchannel with hyperbolic contraction were experimentally assessed using image analysis techniques, and were subsequently compared with the numerical results. The numerically and experimentally obtained velocity fields were in good agreement, with errors smaller than 10%. Additionally, a nearly constant strain rate was observed in the contraction region, which can be attributed to the quasilinear increase in the velocity along the hyperbolic contraction. Therefore, the numerical technique used was validated due to the close similarity between the numerically and experimentally obtained results. The tested CFD model can be used to optimize the microchannel design by minimizing the need to fabricate prototypes and evaluate them experimentally.

## 1. Introduction

Blood consists of a suspension of cells, proteins, and ions in plasma, containing red blood cells (RBCs), white blood cells, and platelets that make up 46% of the volume of the solution. The RBCs represent approximately 45% of the blood volume, strongly influencing the flow in the microcirculation. Healthy RBCs have a biconcave shape with a larger diameter of 7–8 µm and a thickness of 1–3 µm, when in an isotonic medium (plasma or saline solution), and a mass density of about 1.08 × 10^3^ kg/m^3^ [1,2,3,4,5,6,7,8,9].

The study of RBCs can provide physiological and clinical information about a patient through their deformation. Several researchers have correlated the stiffness and deformation of RBCs with various diseases, such as type II diabetes [10], sickle cell anemia [11,12], chronic renal disease [13], and malaria [10,14,15], which can affect blood flow due to changes in viscosity caused by RBC stiffness. Therefore, pathological RBCs, being more rigid, suffer more difficulties in passing through a microcapillary because they cannot deform easily [9,16,17,18,19].

The main function of blood is to transport oxygen, nutrients, waste products, and heat throughout the body. Therefore, oxygen-rich blood leaves the heart at high speed and pressure through the arteries, then travels to the arterioles, and eventually to the capillaries where pressure and speed are reduced. Returning to the heart, blood travels through the venules, then through the veins, and back to the heart to start a new cycle [4,17,20].

Blood in large arteries is often considered a homogeneous fluid for the purpose of modelling, because the diameter of blood vessels is much larger than the length scale of the cellular components of blood. In such case it is characterized as a fluid with continuous viscosity, ignoring the effect of blood constituents [2,21]. As one moves from the arteries to the capillaries (2 to 10 µm in diameter), pressure and velocity decrease. In the capillaries, the Reynolds number (Re) reaches values smaller than 1, where viscous forces dominate over inertial forces. Therefore, the effects that occur at the microscale must be considered, taking into account the multiphase properties of blood (a non-Newtonian fluid) during flow, such as the Fahraeus–Lindqvist effect [2,8,22,23]. For typical human hematocrits (Hct) in the range of 40–45%, it is known that blood viscosity depends of shear rate Therefore, blood behaves as a non-Newtonian fluid, and this property also depends on temperature, Hct, and on the RBCs being healthy or diseased [2].

Microfluidics is the science and technology that has the ability to manipulate and process small amounts of fluids in microscale devices. The application of this technology allows the saving of reagents, the use of smaller samples, and the separation and detection of phenomena, with higher resolution and sensitivity, reducing costs and time. Thus, this technology presents an attractive approach compared to conventional experimental techniques [9].

The determination of human RBCs’ deformation can be performed using various techniques. Micropipette and optical tweezer techniques involve the shear and extensional effects, with most studies focusing only on the shear effect. These techniques are laborious and time-consuming, involving manipulative processes and direct contact between blood components and the tools used [11,24,25]. However, advances in microfabrication have allowed the manufacturing of microfluidic devices capable of visualizing, measuring, and controlling the deformation of the RBCs, allowing for the observation of the effects that occur in the microcirculation through sudden contractions or expansions. Microfluidic devices allow one to control the extensional effect, either separately or simultaneously with the shear effect [13,24]. Furthermore, extensional flow characterization can be performed using e.g. cross-shaped channels [3,26] or contractions, with the use of hyperbolic channels highlighted in several studies [11,27]. Table 1 presents the main characteristics of different types of channels, with a particular emphasis on hyperbolic channels (which provide the greater control and manipulation of the cells passing through the microchannel) [27,28].

Hyperbolic microfluidic devices can be divided into two categories depending on the cellular deformation mechanism. Constriction channels have a local dimension smaller than that of the cells being studied, which means that the cells undergo deformation through the walls of the microchannel, requiring a uniform size of the cells being studied (very large cells block the channel/very small cells flow through the channel without deformation), and they may have variable friction and adhesive effects, which are difficult to quantify. On the other hand, wide cross-sectional channels allow for fluid-induced deformation, without cell-wall interaction, allowing the processing of heterogeneous cell populations, and clogging is unlikely to occur [27].

Generally, in devices that have hyperbolic microchannels followed by an abrupt expansion [11]. the convergence region induces a constant deformation rate resulting from a linear increase in velocity along the contraction and high shear stress, which reaches its maximum at the contraction, showing low velocity and shear values at both the inlet and outlet of the contraction. These characteristics make hyperbolic microchannels very suitable for validating numerical simulations [29] and to measure deformation in a controlled manner, allowing the deformability of not only blood cells [10,11,13,20,25,30] but also other kinds of fluids such as blood analogs [31,32,33]. The uniform shear rate promotes a well-controlled way to measure cell deformation and fluid viscosity and, in this way, to assess single-cell mechanical stresses due to fluid extensional flow [11,13,24,27,34].

The purpose of this work is to compare the flow, trajectories, and velocity profiles of particles and RBCs through a hyperbolic microchannel using both experimental and discrete phase numerical studies.

## 2. Materials and Methods

### 2.1. Experimental Setup

The working fluid used in this study was composed of Dextran 40 (Dx40) with 1% human RBCs, collected from the blood of a healthy adult volunteer and subsequently processed to separate the RBCs. The density and dynamic viscosity of Dx40 were 1046 kg/m³ and 4.5 × 10^−3^ Pa s, respectively [25].

**Figure 1:**
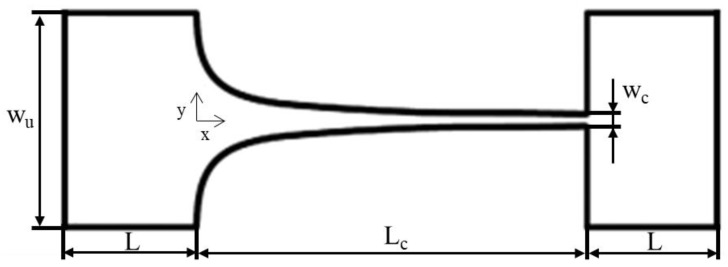
Schematic representation of the hyperbolic contraction microchannels used. The dimensions of the different parts are indicated below in Table 2.

Hyperbolic contraction microchannels were produced in polydimethylsylane (PDMS) via soft lithography, based on an SU-8 photoresist mold [25,35]. The characteristic geometry of the contraction used in this study is shown in Figure 1.

**Figure 2:**
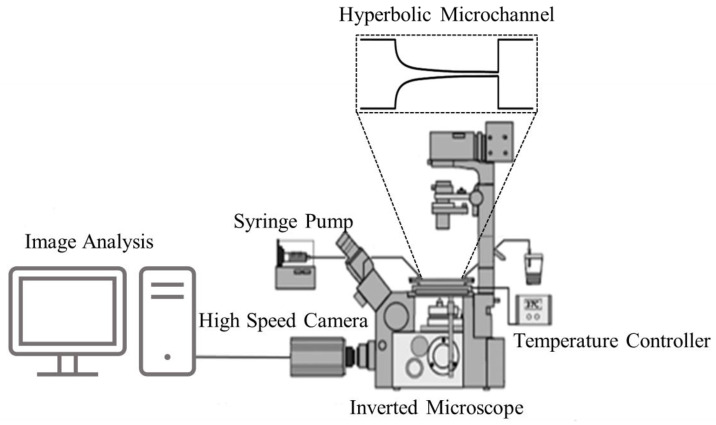
Experimental setup including an inverted microscope, high-speed camera, syringe pump, and thermo plate controller.

The experimental models were placed under an inverted microscope with temperature control, and the flow rate was controlled by a syringe pump ranging from 7.9 × 10^−3^ to 0.265 mL/h. Images of the RBC flow were captured using a high-speed camera (Phantom v7.1, Vision Research, Wayne, NJ, USA) in three regions: the beginning of the contraction, the end of the contraction, and the expansion region. The relevant experimental parameters are summarized in Table 2, and the experimental setup is shown in Figure 2.

**Figure 3:**
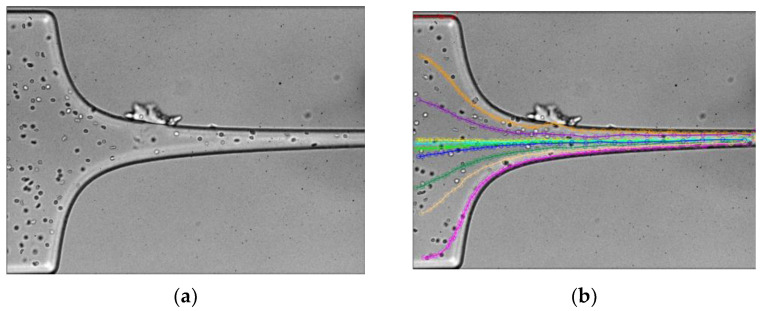
Images analysis sequence: (**a**) brightness and contrast improvement and (**b**) particle tracking.

To determine the trajectories and velocities in the contraction region and downstream of its image analysis, techniques were employed. The trajectory of a selected set of RBCs was manually tracked using the MTrackJ plug-in of the ImageJ 1.51k software to determine their velocity profiles. Cell sampling was performed at various positions along the y-axis, upstream of the contraction, to obtain different particle trajectories and velocity profiles. Figure 3 shows the sequence of steps performed in Image J, where an automatic video adjustment of brightness and contrast was first performed, followed by particle tracking [25].

### 2.2. Numerical Setup

The geometry of the microchannel used in the numerical simulations was selected to closely match that of the experimental flow studies (Figure 1). The lengths of the input and outlet channels (*L*) were set at 500 μm, as well as having the same width (*W*_u_) and depth (*h*) of 406 µm and 60 µm, respectively. The remaining dimensions of the channel are indicated in Table 2.

**Figure 4:**
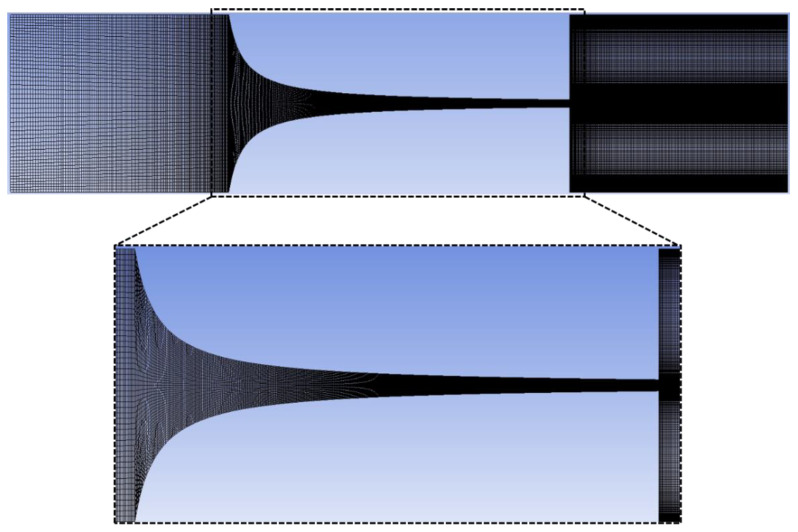
Mesh representation used in numerical simulations.

To configure the numerical model, a hexahedral mesh of almost 2.5 M cells was generated using ANSYS^®^ software’s (2022 R2)merging feature (Figure 4). The mesh utilized exhibits characteristics of being nonorthogonal, nonuniform, and structured.

An analysis was conducted to assess the lack of impact of the mesh on the outcomes, and the findings are detailed in Table 3. The quality of the mesh was validated by using the aspect ratio, skewness, and orthogonality.

**Figure 5:**
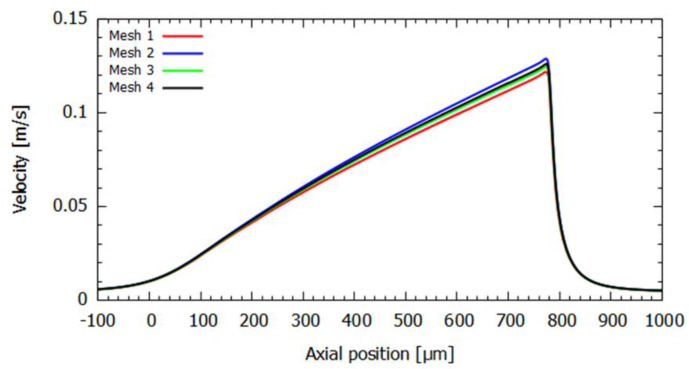
Graphical representation of the velocity performance along the longitudinal axis for different tested meshes at the highest flow rate.

Through the analysis of Table 3, it can be observed that all developed meshes fall within the recommended values for the three parameters studied. Since a detailed flow analysis was desired, mesh 3 was selected due to its high element density in the contraction and outlet regions. Furthermore, the velocity values at the contraction outlet remain relatively constant in the meshes (mesh convergence) or the maximum flow rate tested (Q = 0.265 mL/h), allowing for a more accurate analysis of the microfluidic phenomena (Figure 5).

Additionally, to enhance the validation process beyond the use of mesh parameters (see Table 3) and visual aids, the velocity profile was plotted along the longitudinal axis of the microchannel (see Figure 5). This graphical representation allows one to observe that all meshes yield similar results, except for meshes 1 and 2 (see Table 4), where the error of the trend line in the linear part (hyperbolic region of the microchannel) is higher. Therefore, it can be concluded that mesh 3 is a suitable choice to conduct simulations in Fluent.

ANSYS^®^ Fluent software was used to perform the CFD simulations with the discrete phase model (DPM). The DPM constitutes a numerical modeling approach that is used to simulate the behavior of particles in a continuous medium (fluid). It is used to verify and understand the movement, trajectories, and behavior of particles within a specific device, such as microfluidic devices. Its main advantages include the capability to study the trajectories and behaviors of particle flow in microfluidic devices, as well as complex interactions, for example, among blood cells. However, it is a computationally intensive method compared to conventional approaches and requires rigorous control in the discretizations between different phases (assumptions and simplifications) [37,38].

Accordingly, the flow was computed by solving the continuity and Navier–Stokes equations (Equations (1) and (2), respectively):

(1) ∇·u=0

(2) $$\rho \left[\frac{\partial u}{\partial t}+\left(u.\nabla \right)u\right]=-\nabla p+\nabla \cdot \tau$$

where *u* is the velocity vector, ρ is the density of the fluid, *p* is the pressure, *t* is the time, and τ the total extra stress tensor. Since Dx40 is a Newtonian fluid, the fluid was modeled as an incompressible Newtonian fluid, where density and dynamic viscosity were assumed to be 1046 kg/m³ and 4.5 × 10^−3^ Pa s, respectively.

The particle trajectories were calculated by integrating the forces that are applied on the particle on the basis of a Lagrangian specification frame of reference. Thus, the trajectory is predicted from the balance between the inertial forces and the forces applied on the particle (Equation (3)):

(3) $$m_{p}\frac{\partial u_{p}}{\partial t}=m_{p}\frac{u-u_{p}}{\tau_{r}}+m_{p}\frac{g\left(\rho_{p}-\rho \right)}{\rho_{p}}+F$$

where $m_{p}$ represents the particle mass, $u_{p}$ the particle velocity, $\rho_{p}$ the particle density, *F* an additional force, $m_{p}\frac{u-u_{p}}{\tau_{r}}$ the drag force, and $\tau_{r}$ the particle relaxation time.

Furthermore, the rotation of the particle in the fluid was also considered because it can have a significant influence on particles with high mass and large size and with a high moment of inertia. Thus, an additional ordinary differential equation (ODE) for the particle’s angular momentum was solved:

(4) $$I_{p}\cdot \frac{\partial \omega_{p}}{\partial t}=\frac{\rho }{2}\cdot {\left(\frac{d_{p}}{2}\right)}^{5}C_{\omega }\cdot \left|\Omega \right|\cdot \Omega =T$$

where $I_{p}$ represents the moment of inertia, $\omega_{p}$ the particle angular velocity, $d_{p}$ the particle diameter, $C_{\omega }$ the rotational drag coefficient, *Ω* the relative particle–fluid angular velocity, and, *T* the torque applied to a particle in a fluid domain.

The no-slip wall boundary condition and the validity of the continuum hypothesis are well established in micrometer-scale Newtonian fluid flows [34]. The similarity between the experimental and numerical results shows that the conditions previously applied are appropriate.

The previously presented governing equations are solved using the coupled scheme, where a pressure–velocity coupling algorithm is implemented. Since the present work focuses on steady-state calculations, the temporal derivative is discretized using a first-order implicit Euler scheme [34].

With respect to boundary conditions, a uniform velocity profile was considered in the region upstream of the contraction for the input. On the other hand, the outlet downstream of the contraction was defined as an outflow boundary condition, which implies zero-gradients for the velocity and stress components and a constant pressure gradient at the channel outlet (*L* = 500 µm) [34]. The no-slip wall boundary condition was imposed on the walls.

Additionally, the Reynolds number was calculated from Equation (5).

(5) $$Re=\frac{\rho \cdot {\left\langle V_{z}\right\rangle }_{u}\cdot w_{u}/2}{\mu }=\frac{\rho \cdot Q}{2\cdot h\cdot \mu }$$

where *Q* represents the volumetric flow rate and 〈*V*_z_〉_u_ is the average velocity in the upstream channel. This dimensionless number measures the ratio between inertial and viscous forces, thus being helpful in predicting the flow pattern in different situations.

Taking into account the conditions and geometry, with Dx40 as the working fluid, an Re = 0.054 *Q* is achieved, with *Q* expressed in ml/h.

## 3. Results and Discussion

### 3.1. Fluid Flow within the Hyperbolic Contraction 

**Figure 6:**
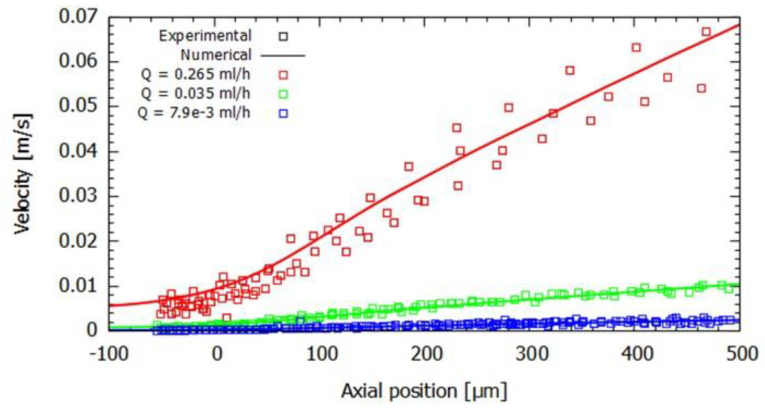
Evolution of the RBC velocity as a function of the axial position in the contraction region for the flow rates studied. Symbols and lines represent experimental and numerical results, respectively.

For the three flow rates under study, the RBC velocities and strain rate values were tracked along the axis of the microchannel up to a length of 500 μm. The nearly linear increase in velocity along that axis is independent of the flow rate under study, as can be observed in Figure 6. The axial velocity profile was determined for each flow rate using the average of at least three particles. As expected, the increase in flow rate results in an increase in velocities in the microchannel. As the velocity increases nearly linearly for each flow rate, the strain rate, calculated through the slope $du_{x}/dx$, where $u_{x}$ is the axial velocity of the particle, will remain approximately constant inside the contraction. Furthermore, the magnitude of the strain rate was observed to increase with the flow rate (Table 5), resulting in values in the range of 4.116–120.824 s^−1^ for experiments and 4.402–121.296 s^−1^ for numerical simulations. 

The strain rate values for each initial central longitudinal position are reported in Table 5 for each flow rate, where it can be observed that the coefficient of determination, *R*^2^, shows a very close fit to the regression model used, which confirms the good linear approximation of the RBC velocity within the defined domain. Furthermore, the error values are below 10%, indicating good agreement between the numerical model and the experimental data. An increase in the deviation error is observed for the lower values of the flow rate, which could be related to the particle selected to carry out the particle tracking from the experimental recorded videos.

**Figure 7:**
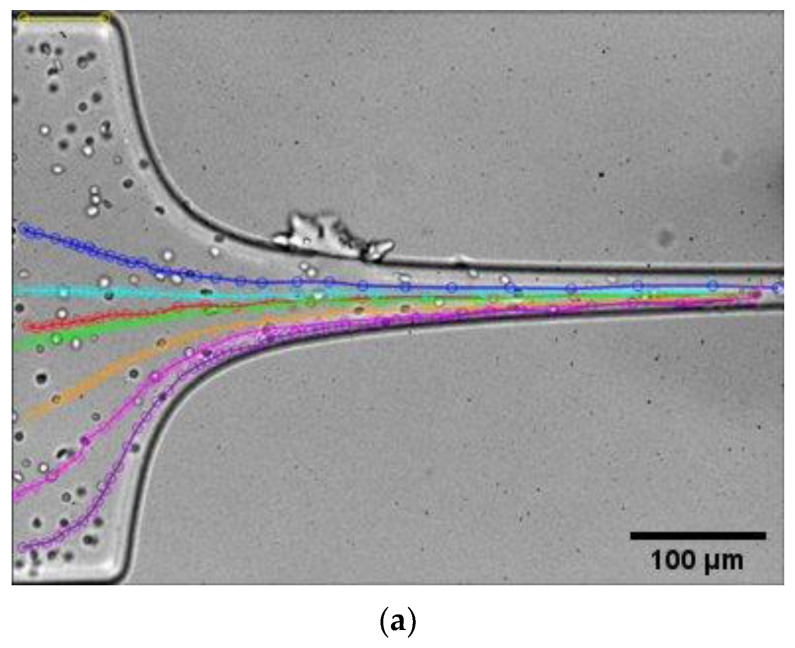

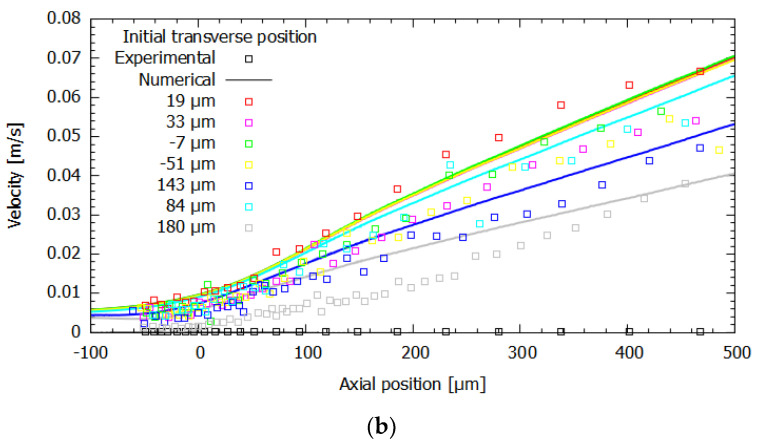
Effect of RBC starting initial coordinate (y-axis) on the axial velocity profile, for experimental (symbols) and numerical (lines) data: (**a**) experimental tracking of RBCs and (**b**) velocity of RBCs as a function of axial position.

The results concerning the influence of the starting position of the RBCs and their velocity change within the contraction are shown in Figure 7. As can be seen in Figure 7b, the resulting flow velocity increases as the distance from the axis of the microchannel decreases in the transverse direction. Particles with initial positions closer to the longitudinal axis of the microchannel exhibit velocities higher than those further away, mainly due to not being subjected to the effects of shear stress present on the walls of the microchannel. Furthermore, it can be observed that once more, the velocity closely follows a linear change with axial distance traveled for all experimental positions and increases as we progress towards the contraction and as the width of the device decreases. Thus, it can be inferred that the velocity will reach its maximum at the end of the contraction, just prior to the rapid expansion of the microchannel. Nevertheless, the experimental and numerically assessed values for the velocity do not match for the same inlet position, which may be due to the fact that the numerical simulation assumes that the particles are located at the half-height of the channel, while in the data from the experiments, this information is not available since the tracking image system only acquires information in two dimensions, irrespective of the RBC momentary applicate coordinate (z-axis position). Furthermore, the RBCs are deformable cells and the dynamics of RBCs in shear flow are different from rigid particles, which was not taken into account in the numerical model.

### 3.2. Fluid Flow at the End and Downstream of the Hyperbolic Contraction 

**Figure 8:**
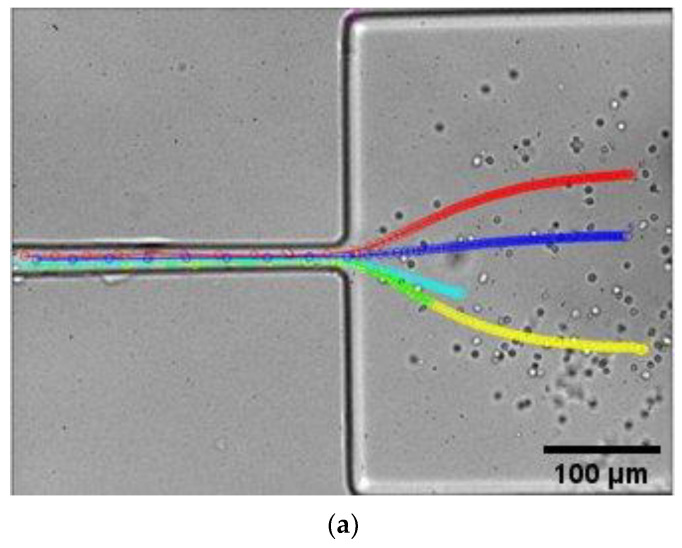

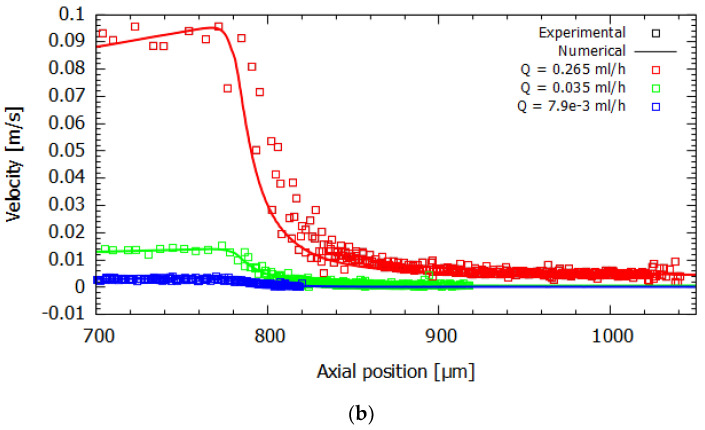
Influence of flow rate on the axial velocity profile determined experimentally (symbols) and numerically (lines) at the end and at the hyperbolic contraction downstream: (**a**) experimental particle tracking and (**b**) particle velocity profile as a function of axial position.

Figure 8 shows both the numerical and experimental results at the end of the contraction, where the maximum velocity is reached. Following the region of maximum contraction and consequent maximum velocity, a drastic drop in velocity is observed due to the abrupt expansion that takes place at the hyperbolic contraction downstream. The simulation accurately captures this phenomenon, as the experimentally assessed particle positions closely follow the trend line resulting from the numerical simulation.

### 3.3. Tranverse Velocity Profiles within the Hyperbolic Contraction

**Figure 9:**
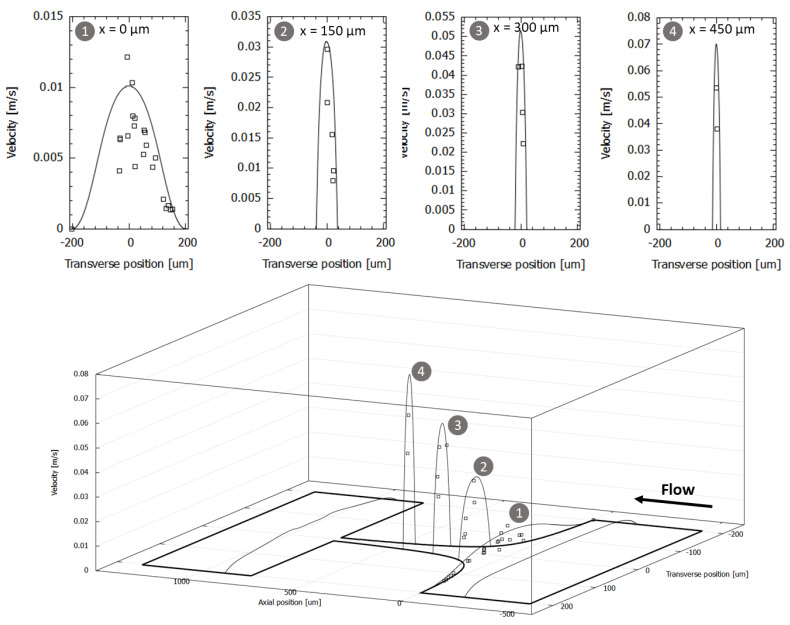
Comparison of the velocity profiles in the midplane of the hyperbolic microchannel for different axial positions and Q = 0.265 mL/h. Experimental and numerical data are represented as symbol and line, respectively.

The experimental and numerical velocity profiles were determined in the flow direction at various axial positions and at the half-height of the channel for the numerical case, considering a flow rate value of 0.265 mL/h. Upstream of the contraction plane, at x = 0, the velocity profiles resemble those of a developed flow, as can be seen in Figure 9. However, as we progress towards the contraction, the fluid tends to be displaced towards the microchannel central region, resulting in an increase in velocity close to the main axis and a decrease near the walls. As previously observed, the maximum velocity is reached along the center line of the microchannel and increases as we move through the contraction. By analyzing the results for each transverse plane (see Figure 9), it can be seen that initially, the agreement of the results is low for *x* = 0 µm, but when the longitudinal position increases, the agreement between the experimental and numerical results is high. The initially low agreement may be due to the track of cells that were not in the center region of the channel all the time, due to the high-volume illumination performed with our high-speed video microscopy system. 

## 4. Conclusions

The study of the hemorheological properties of red blood cells (RBCs) is of significant clinical importance, as it provides direct and indirect information on various pathologies. As such, the use of microfluidic devices allows the simultaneous study of multiple cells in a single test, such as their deformability and motion at different flow rates, to validate the parameters under study.

In this study, we compared experimental microfluidic and in silica numerical results to analyze the behavior of RBC that flows in a hyperbolic microchannel, with a particular focus on velocities and deformation rates. The experimental data revealed that the RBC velocities reach a maximum value at the end of the contraction, as expected, while the strain rate remains constant throughout the contraction. Furthermore, good agreement between the numerical and experimental studies was verified, with relative errors in the region of linear dependence of the velocity less than 10%, demonstrating a very high level of consistency between experimental and numerical data. In the outlet region, an abrupt drop in velocity was observed for the numerical model, once more in accordance with the experimental results. Furthermore, it was observed that RBCs tend to have a linear velocity increase along the contraction. The experimental data were used to validate the numerical model, which could prove useful for the acceptance of discrete phase methods in studies involving microfluidic devices. 

In future studies, it is expected to validate this method for more complex flows that occur in microvessels and microfluidic devices, such as the cell-free layer and the Fåhræus–Lindqvist effect. Furthermore, this method is intended to be the basis for validating meshless simulation methods, where the simulation and study of phenomena happening at a micro/nano scale level is the most natural choice. 

## Figures and Tables

**Table 1:** Characteristics of different types of microchannels [28].

Channel	Pros	Cons
Cross-shaped Channel	Bounded flow enhances flow stability	Trajectory focusing is required
	Stagnation point-trapped cells are ideal for observation	Only one cell is present at a time within the stagnation zone
	The extensional rate magnitude can be easily regulated	There exists a restricted area of purely extensional flow
Sudden/smooth Constriction	Easily producible	There exists a restricted area of purely extensional flow
	High-throughput approach	Trajectory focusing is required
	The magnitude of the extensional rate can be easily controlled	At high flow rates and pressures, it is difficult to perform flow measurements and leakage is likely to happen
Hyperbolic Constriction	The magnitude of the extensional rate can be easily controlled and manipulated	There exists a restricted area of purely extensional flow
	Nearly constant strain rate	Trajectory focusing is required
	High-throughput approach	At high flow rates and pressures, is difficult to perform flow measurements and leakage is likely to happen

**Table 2:** Experimental parameters: channel dimensions, working fluid, and image acquisition settings.

Parameter	Symbol	Dimensions
Upstream and downstream widths	w_u_	406 µm
Minimum width of contraction	w_c_	17 µm
Length of contraction	L_c_	780 µm
Microchannel depth	h	60 µm
Inlet and outlet lengths	L	500 µm
Flow rate	Q	(7.9 × 10^−3^, 0.035, 0.265) mL/h
Mean diameter of human RBC at rest	d_p_	8 µm
Dx40 shear viscosity	μ	4.5 × 10^−3^ Pa s
Dx40 density	ρ	1046 kg/m^3^
Hematocrit of the working fluid		1%
Temperature		37 °C
Camera frame rate		4800–13,000 fps
Frame interval		77–208 µs

**Table 3:** Characteristics of the meshes used in this study [36].

Characteristics	Mesh				Advised Value
	1	2	3	4	
Nodes	97,125	524,271	1,948,023	2,524,802	---
Elements	80,520	468,000	1,832,000	2,386,560	---
Aspect Ratio	6.022	6.180	4.327	4.671	<100
Skewness	3.398 × 10^−2^	2.537 × 10^−2^	1.980 × 10^−2^	2.347 × 10^−2^	<0.75
Orthogonal Quality	0.982	0.988	0.992	0.989	>0.70

**Table 4:** Characteristics of the tested meshes for the hyperbolic microchannel [36].

Parameter	Mesh Used (3)	Mesh 1	Mesh 2	Mesh 4
Strain Rate	149.74	145.51	153.61	151.01
Error	---	2.82	2.58	0.85

**Table 5:** Strain rate values for each central longitudinal initial position for experimental and numerical tests.

Flow Rate [ml/h]	Experimental			Numerical	Error (%)
	Strain Rate	R^2^	Mean Strain Rate	Strain Rate	
0.0079	3.970	0.971	4.116	4.402	6.950
	4.633	0.986			
	3.744	0.971			
0.035	18.528	0.993	18.000	18.762	4.233
	17.806	0.994			
	17.666	0.996			
0.265	127.537	0.997	120.824	121.296	0.390
	110.152	0.996			
	124.784	0.997			
